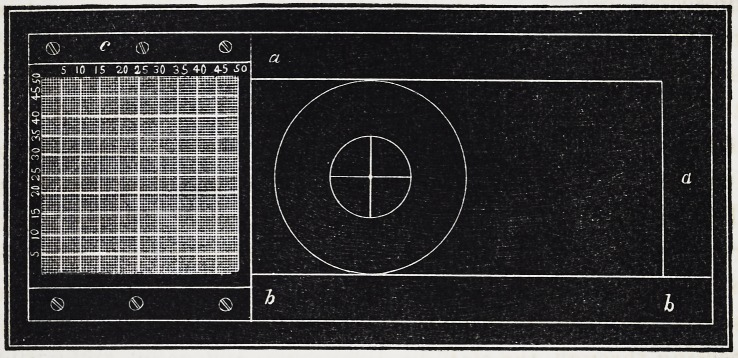# Report of the Committee Appointed by the Microscopical Society for the Purpose of Ascertaining the Most Convenient Form of Finder for Indicating the Position of Objects under the Microscope

**Published:** 1857-01

**Authors:** George Jackson, Chas. Brooke, F. H. Wenham


### ARTICLE XII.

*Report of the Committee appointed by the Microscopical Society for the purpose of ascertaining the most convenient form of Finder for indicating the position of Objects under the Microscope.*—Read June 25th, 1856.

AN apparatus for registering the position of any number of minute objects contained in a slide, and for readily finding any one of them, either under the same microscope, or any other, has for some time been a desideratum with observers; and various plans have been proposed. The council of the Microscopical Society have, therefore, thought fit to appoint a committee to consider the subject, and to suggest some means of accomplishing the object, of such a simple and inexpensive character as would merit universal adoption.

The committee have held three meetings, besides having had communications by letter, and they now proceed to lay before the society the result of their deliberations.

They consider that a finder, in order to be universally adopted, should possess the following properties:

1st. It should be applicable to any microscope, whether furnished with stage-movements or not; and it should not preclude the use of these movements.

2d. It should not require fresh labels to be placed on the slides, or any mark or index to be made on them.

3d. It should not be necessary to remove the slide or finder for the registering or finding of every separate object.

4th. The divisions on the index should be easily read.

5th. It should allow the microscope to be used in the inclined position; and—

6th. It should be cheap, and simple enough to be constructed by any one possessing a moderate amount of mechanical skill.

In the plans which they recommend for carrying out these requirements, the committee lay no claim to originality; for they have merely selected materials from what has been already proposed, and arranged them in a modified form. They are, therefore, free from the prejudice which an inventor would naturally feel in favor of his own ideas.

Whatever modification an individual may contrive for his own convenience, in the form of the finder, the same standard of measurement should be adopted; and the measurements or distances of objects must be taken relatively to the same fixed point. This point should by no means have reference to any part of the microscope (which would be fatal to a *universal* system,) but should be considered as a point *in the slide itself*, upon which the object is mounted.

The slides recommended by the Microscopical Society (three inches by one) are now so generally used that it is not worth while to propose a method of meeting exceptional cases; therefore the above starting point may, under all circumstances, be represented by the intersection of too crossed lines taken as perpendiculars, one and a half inch from one end of the slide, and half an inch from one side. As it would be difficult and troublesome to rule this cross on every slide, it is preferable to have one plain slide as a standard, with the cross occupying the position above named. The measured distance being taken strictly from *one* end and *one* side, the corner common to them should be marked with an arrow-head, in order to avoid the error that would be occasioned if the glass were not cut of the *exact* dimensions, and the wrong end were used in the adjustment. These lines should be ruled with a diamond point, filled with plumbago, and covered with thin glass.

It now remains to explain the application of this fundamental starting point for rendering all forms of finders universal.

First, with respect to microscopes without stage movements. Fig. 1, *a*, *a*, is a carrier, made either of metal or wood, whose outside dimensions are three and a quarter inches long by one and a half wide. Along the lower margin is fixed a raised edge, one quarter of an inch broad, for the slide to rest against. There is a hole one inch in diameter in the carrier, the distance of whose centre is one and a half inch from the right-hand end.

On the left-hand end is fastened to the raised edge, or slip, a piece of brass, *c*, one and a quarter inch square, having a sufficient space beneath it to allow the thickest slide to pass under and abut against a stop at the end; by this means an inconvenient length of the carrier is avoided. The upper plane surface of this plate contains the index, which may be printed on enamelled paper, and contains one square inch, divided by lines at distances of one-fiftieth of an inch, crossing each other at right angles. The two lines which cross the centre each way are considerably thicker than the rest, and are numbered 25 (being half the number of the divisions;) and the intersection of these is always the starting point for making the adjustments. Every fifth line from this is rather less in thickness, and is numbered at the side; the intermediate ones being as fine as may be convenient for distinct observation. The exact position in which the paper index is pasted on is not of material consequence, provided it lies square with the carrier.

The method of using this finder is as follows: First take the *standard*, and place its marked end against the abutment under the index-plate of the carrier, and see that it rests upon the bottom ledge. Then place them both together under the microscope, with an object-glass attached. By sliding up or down the vertical moving straight edge (with which every plain stage should be fitted,) and by shifting the carrier sideways against this edge, bring the cross into the centre of the field. A movable index, or hand, is now made to point to the centre, or crossing of the thick lines on the index-plate, and there fixed fast. This hand, or pointer, is simply a thin, flat piece of brass, turned round like a hook or staple, having the longest limb ending in a point, and the shortest slotted, to be clamped to the under side of the fixed stage-plate, by means of a milled-headed screw passing through the slot.

The longitudinal adjustment of the pointer is performed by thrusting it endways, and the transverse by turning it sideways. Now look through the microscope again, to ascertain if the cross on the standard is still in the centre of the field, and coincident with the adjustment of the pointer. If so, remove the standard, and replace it by the slide containing objects whose position is either to be registered or found. If the pointer is not in the way when not in use, it may remain fixed; and readjustment by the standard will be unnecessary except for occasional verification. It is now evident that the centre of the field is represented by the pointer on the index-plate at an invariable distance; therefore, if the carrier be always moved in straight lines at right angles, the pointer will indicate the latitude and longitude of the object under the microscope at the time.

Although the centre of the slide is taken as the most convenient point for adjusting the index, it is best to consider the lower horizontal line on the index-plate as the equator, and the left-hand perpendicular one as the first meridian; by which means all the latitudes will be north, and the longitudes east; and, if the first figure be invariably appropriated to the latitude, the registration will be very simple. Should it be necessary to register smaller quantities than the fiftieth of an inch, the amount may be estimated with sufficient accuracy, and added as a decimal figure; and this will be found much easier in practice than the reading of very fine divisions.

In registering a large cabinet of *Diatomaceæ*, *Desmidieæ*, &c., it will only be necessary to number the slides, and to enter on a printed list of these objects, the number, latitude, and longitude where they may be found. By these means any specimen may be placed under the misroscope in less than a minute.

The finder may be applied to microscopes having stage-movements by merely laying the carrier against the horizontal straight edge on the top plate; and, after fixing the rotary plate (if there be one) by a pin passed into a hole drilled through this plate and the next, the adjustments already described may be made, using the milled heads for the purpose. In those microscopes which have the longitudinal and perpendicular sliding plates divided into fiftieths of an inch, the principle of a universal finder may be applied in a very simple manner. The rotary movement being fixed in the above mode, there must be a stop, consisting of a strip of plate brass, with one end turned up, to form an abutment, and the other slotted and passing under the head of a screw in the top plate, so as to move with sufficient friction to retain its position.

The gradual scales should then both be set to read 25, and the standard placed on the stage. Then bring the cross into the centre of the field by shifting the stop laterally, and moving the top plate, or straight edge, perpendicularly, taking care to keep the standard hard against both its bearings. When this has been effected the standard may be removed, and, if any slide be placed in the same position against the stop and ledge, the objects in it may be registered and found by means of the graduated scales, freely using the stage-movements for the purpose, but being careful not to shift the top plate or sliding stop.

This form of finder is to be recommended where rectilinear stage-movements exist, on account of its simplicity. A substitute for the graduated scales may be obtained by cutting off the edges from two of the square index-plates here described, and cementing them in the most convenient situations.

Another method would be to fasten one of the entire square scales on the top plate of the microscope ; but this would involve the necessity of a pointer, as in the case of using the carrier.

Having now described a finder that can be used either with the most simple form of microscope, as a temporary addition to the ordinary stage-movements, or as permanently incorporated with them, we by no means desire arbitrarily to assert that these plans are the best that can be devised. The subject is still open to improvement; but, whatever modification is adopted, in performance of our duty we strongly urge the necessity of basing them all upon *one similar standard of measurement,* taken from a *fixed point on the object-slide itself.* If this be done, objects registered by one observer may be found by another, though each may use a finder of a different form ; the difference affecting merely convenience in using.

There is one observation arising from this subject, although not strictly a portion of it, which the committee wish to make. If, by reason of defective workmanship, or errors caused by the use of adapters, different object-glasses are not in the same line of centre, a readjustment by means of the standard will be required on changing the objective. We, therefore, call attention to the great convenience it would be to all users of the microscope if every maker would adopt the same pattern for a screw, a proper steel gauge being provided for the purpose. At present it not unfrequently happens, on applying another object-glass to a microscope, that it has to be built upon a system of three or four adapters, to the manifest detriment of its performance. If all our principal makers will acquiesce in this, we shall be most happy to give our assistance in establishing the best form of screw.

As our endeavor has principally been to determine the means by which a uniform system of registration may be obtained, we have not thought it necessary to make any critical inquiry as to the respective merits or defects of the plans already published; we have borrowed freely from them; and we have also received valuable practical suggestions from Professor Quekett and Mr. Hislop, for which we here tender our thanks.

(Signed) GEORGE JACKSON,
CHAS. BROOKE,
F. H. WENHAM.

## Figures and Tables

**Figure f1:**